# Effects of Family Nurture Intervention in the NICU on Theory of Mind Abilities in Children Born Very Preterm: A Randomized Controlled Trial

**DOI:** 10.3390/children9020284

**Published:** 2022-02-18

**Authors:** Morgan R. Firestein, Michael M. Myers, Katherine J. Feder, Robert J. Ludwig, Martha G. Welch

**Affiliations:** 1 Department of Psychiatry, Columbia University Irving Medical Center, New York, NY 10032, USA; mmm3@cumc.columbia.edu (M.M.M.); mgw13@cumc.columbia.edu (M.G.W.); 2 Department of Pediatrics, Columbia University Irving Medical Center, New York, NY 10032, USA; rjl2128@columbia.edu; 3 Yale University School of Medicine, New Haven, CT 06510, USA; katherine.feder@yale.edu

**Keywords:** premature, neonatal, neonatal intensive care, maternal separation, social cognition

## Abstract

Preterm infants are at risk for socioemotional deficits, neurodevelopmental disorders, and potentially theory of mind (ToM) deficits. Preterm infants enrolled in a randomized controlled trial in the neonatal intensive care unit (NICU) received Standard Care (SC) or Family Nurture Intervention (FNI). Children (N = 72; median age 61.8 ± 2.6 months; FNI: 35 (55%), SC:2 9 (45%)) completed a ToM task, of whom 64 (54% male; born to White (43.8%), Black (18.7%), and Hispanic (25.0%) mothers) contributed to this analysis. FNI and SC infants born extremely preterm to very preterm differed significantly: 78% (14 of 18) of FNI children passed vs. 30% (3 of 10) SC children (*p* = 0.01, effect size = 1.06). This large effect size suggests that FNI in the NICU may ameliorate deficits in social-cognitive skills of extreme to very preterm infants by school age.

## 1. Introduction

Theory of mind (ToM) is a complex social-cognitive ability that allows one to recognize that others have their own mental states including thoughts, feelings, beliefs, and desires that are different than his or her own [1]. The ability to employ ToM requires complex coordination of areas of the brain necessary for cognitive and socio-emotional intelligence and is likely crucial for establishing positive social relationships with others [2,3,4]. This capacity typically emerges between four and five years of age, though instability in ToM at this age has been noted [5,6,7]. Data provided by functional MRI brain imaging have led to a distinction by some researchers between ‘cognitive ToM,’ ‘cognitive affective empathy,’ and ‘affective empathy’, with each being associated with different brain regions [8]. Recent studies of children with ToM deficits have identified various brain regions associated with the delayed onset of ToM [9]. Among the mechanisms proposed to account for ToM is the complex coordination of areas of the brain necessary for establishing ‘emotional intelligence’ [10].

Autism spectrum disorder (ASD) is a pervasive neurodevelopmental disorder characterized by impairments in social interaction and communication, as well as restricted or repetitive behaviors and interests [11]. It has been widely observed that children with ASD, even those older than five years of age, fail to employ ToM [12]. ASD is a highly heterogenous condition and our understanding of the etiology underlying ASD is limited. One well-established risk factor for ASD is premature birth [13,14,15]. The mechanisms underlying the long-term neurodevelopmental consequences of preterm birth can be difficult to discern as prematurity is often associated with suboptimal aspects of both the prenatal and early postnatal environments. Hospitalization in the neonatal intensive care unit (NICU) involves routine exposure to painful procedures, increased sensory stimulation, and repeated maternal separation [16,17,18]. By 18–24 months of age, 25% to 40% of preterm infants meet the criteria for an ASD evaluation, and the overall prevalence of ASD among children who were born prematurely is approximately 5% [13,15]. Preterm infants are more likely to exhibit deficits in social-emotional skills, language and communication, and cognitive domains including attention, inhibitory control, and working memory [19], all of which are likely recruited to employ ToM [20].

In an effort to identify biological and developmental antecedents of ToM, studies have begun to examine the effects of premature birth on the development of ToM [21,22], though the literature is inconsistent. One study found that compared to full-term infants, preterm infants were more likely to fail ToM tasks when tested at three and four years of age, but by five years of age, the performance gap between preterm and full-term infants had closed [23]. In contrast, another study reported significant difference in ToM performance between children born extremely preterm and full-term persisted through 5 years of age [24]. A third study found no difference in performance on a ToM task between preterm and full-term infants as early as four years of age, though the report notes that both groups performed at a below chance (50%) level, bringing into question the validity of the task presentation [25]. At 7–13 years, children born preterm demonstrated reduced activation and connectivity of several brain regions that have been implicated in the ToM network, as measured by magnetoencephalography [26,27]. These results suggest that recruitment of these critical brain regions may be altered in children who are born prematurely and that these changes may persist into adolescence. Collectively, data from studies of prematurely born children remain inconclusive regarding the development time course and neural correlates of ToM.

To study the behavioral, physiological, and social-emotional consequences of preterm birth and subsequent NICU hospitalization, we enrolled a cohort of premature infants into a randomized controlled trial (RCT) of Family Nurture Intervention (FNI) in the NICU [28]. We conducted a randomized controlled trial (RCT) of FNI in a level IV NICU. We enrolled and randomized 150 infants and their mothers into one of two groups. The control group of Standard Care (SC) received standard NICU care. The FNI group received SC plus FNI. The intervention was carried out by trained NICU nurses with the aim of facilitating a strong autonomic emotional connection between mother and infant prior to discharge. To date, significant group differences on measures of infant brain development have been documented including electroencephalographic (EEG) cortical activity at ~35 weeks gestational age (GA) and at term age [29], maternal mental health and caregiving behavior [30,31], infant and maternal autonomic regulation [32,33], and infant neurobehavior [14]. Other NICU-based interventions have been shown to confer long-term neurobehavioral benefits to preterm infants [34,35,36]; however, the impact on ToM development has not been evaluated. The analyses presented here address our hypothesis that FNI would mitigate the previously observed impairments in ToM at preschool age among children born preterm.

## 2. Materials and Methods

Between 2008–2012, 115 mothers of 150 preterm infants born between 26^0^ and 34^6^ weeks GA were invited to take part in a single-center, parallel-group randomized controlled trial of FNI (ClinicalTrials.gov, NCT01439269) in the level IV NICU of NewYork-Presbyterian Morgan Stanley Children’s Hospital of Columbia University Irving Medical Center. Prior to the start of the study, the Columbia University Irving Medical Center Institutional Review Board approved all recruitment, consent, and study procedures. Additionally, written informed consent was obtained from mothers prior to group assignment. Women were eligible to participate if they gave birth to a singleton or twins without significant congenital defects. Additional inclusion criteria included the following: (1) infant birth weight above the 3rd percentile; (2) maternal age of at least 18 years; (3) maternal fluency in English; (4) the absence of prior or current maternal mental illness, addiction, or substance abuse; and (5) the presence of at least one other adult in the home.

The 115 enrolled mothers were randomized to receive either FNI or SC. Study staff were not blind to group assignment because they were responsible for collecting the data that was specific to each group. However, each participant was assigned a de-identified study code that was used for all data entry and analysis. The analyses presented here were performed on data obtained from the children who returned for the 5-year follow-up visit. To minimize potential developmental effects of age at testing, we restricted our analysis to data collected from children who were within 6 months of 60 months CA (4 years 6 months–5 years 6 months). In total, 72 children completed the false belief task, 34 of whom had been assigned to the SC group and 38 of whom had been assigned to the FNI group. Of the 72 children, 64 (43% of the enrolled children) met the criteria to be included in the analyses. Demographic characteristics of these 64 children are included in Table 1. Across both study groups, maternal age ranged from 19 to 55 years (34.7 ± 6.0). Most children were delivered by Cesarean delivery (70.3%) and the GA at delivery ranged from 26 to 34 weeks (31.1 ± 2.11). Overall, 45% of the children were male. Among those who self-reported race and ethnicity, 43.8% self-identified as White, 18.7% self-identified as Black, and 25.0% self-identified as Hispanic. These demographic characteristics were similar to those of the entire enrollment cohort [37].

Trained nurture specialists met with mothers assigned to the FNI group at the earliest time point after delivery to begin administering FNI procedures designed to facilitate mother–infant interaction and emotional connection [28]. Nurture specialists met with mothers assigned to the FNI group for an average of 6.4 hours per week for the duration of the infant’s hospitalization in the NICU. FNI procedures included the following: (1) Scent cloth exchange, in which the mothers were instructed to wear a cloth against her chest while another cloth was placed under her infant’s head. At each visit, the infant received the mother’s cloth and the mother received the infant’s cloth, providing an olfactory exchange between mother and infant. (2) Comfort touch, in which the mother was instructed to touch her infant in the isolette in a gentle, but firm and sustained manner. (3) Vocal soothing, in which the mother was encouraged to speak to her infant in her native language. (4) Skin-to-skin holding, in which the mother was able to hold the infant on her chest once the infant was medically stable enough to be removed from the isolette.

Mother–infant dyads or triads that were assigned to the SC group did not meet with nurture specialists and received the standard care that our NICU-hospitalized infants received independent of the study, such as contact with medical staff, bedside nursing staff, and access to a psychologist and social-worker. Additionally, research assistants met with mothers in both groups once per week throughout NICU hospitalization to administer questionnaires.

At 5 years corrected age (CA), mother–child dyads or triads were invited to participate in a developmental follow-up visit that included the Sally-Anne false belief task to assess ToM. False belief tasks measure a participant’s understanding that another person holds a belief that is incompatible with the reality of the situation, and whether this understanding causes changes in their behavior. The Sally-Anne false belief task, which was first developed by Wimmer and Perner [38] and later revised and simplified by Baron-Cohen et al. [12], addresses two major components of cognitive ToM. First, it examines whether the participant can take the perspective of the scenario’s protagonist and decide based on the protagonist’s knowledge, rather than the child’s own personal knowledge. Secondly, it requires that the participant actively suppresses his or her own knowledge and beliefs regarding situational variables (i.e., the location of the marble), while understanding that the protagonist’s knowledge and beliefs are distinct and different from their own. This particular task is especially difficult because it is not simply that the participant’s knowledge of the situation differs from that of the protagonist, but rather, it requires the participant to understand that the protagonist holds a false belief that is contrary to the actual state of the world.

The task includes three comprehension questions to ensure that the child understands the scenario and one false belief question to determine whether the child can employ ToM. At the beginning of the session, the child was instructed to sit directly across from a research assistant at a table. A small box, a small basket, a marble, and two dolls were placed in front of the child. Following standard procedures, the research assistant introduced the two dolls as “Sally” and “Anne” and the child was asked to identify each doll by name (Naming Question). Participants who failed the Naming Question were corrected and presented with the question again. While holding one doll in each hand, the research assistant acted out and verbally narrated the scene as follows. First, Sally put the marble into either the basket or the box. In order to demonstrate that Sally was leaving the room, the research assistant narrated the action, said ‘Goodbye’ to Sally, and hid the Sally doll under the table. Once she was out of sight, the research assistant visually demonstrated and verbally explained that Anne took the marble out of the first location and placed it into the other so that Anne and the participant, but not Sally, knew the current location of the marble. Next, the box and the basket were covered so that the marble was no longer visible to the child. The research assistant then returned Sally to the desk and asked the participant, “Where will Sally look for the marble?” (False Belief Question). Finally, the research assistant asked two additional comprehension questions: “Where is the marble?” (Reality Question) and “Where was the marble in the beginning?” (Memory Question). No feedback was provided to the participants regardless of their responses following the Reality, Memory, and False Belief Questions. The participants’ responses to each question were recorded by the research assistant. A correct response to the False Belief Question was recorded if the child named or pointed to the original location of the object. An incorrect response was recorded if the child named or pointed to the object’s current location, pointed to both locations, or did not provide a response.

Analyses were performed in SPSS Version 26, R version 4.0.3 and in Systat (Version 13). We performed a Chi-square test of independence to determine whether there was a difference in ToM performance between the two study groups (FNI versus SC). A second Chi-square test of independence was performed to determine whether GA at birth was associated with ToM performance, both across the entire sample and within each study group (FNI versus SC). Infants were categorized into one of two GA groups based on the median GA of the sample: (1) extremely preterm to very preterm (26^0^–30^6^ weeks GA) and (2) very preterm to moderately preterm (31^0^–34^6^ weeks GA). A multiple linear regression model was performed to determine the effect of study group while controlling for relevant demographic variables including the child’s age at testing (continuous), sex (binary: male versus female), mode of delivery (binary: vaginal versus Cesarean delivery), GA at birth (continuous), and number of hours of kangaroo care per week. Only the child’s age at testing was associated with either the predictor or outcome variable at the a priori threshold of *p* < 0.1 and was included as a covariate in the model. Student’s *t*-tests were performed to determine whether the length of stay in the NICU differed significantly between study groups and whether length of stay was associated with ToM performance.

## 3. Results

In total, 72 children (38 FNI, 34 SC) completed the Sally-Anne task at 5 years CA (61.8 ± 2.64 months). Of the 72 children, 64 (35 FNI, 29 SC) correctly answered all three comprehension questions and were therefore included in the statistical analyses presented here. The demographic characteristics between the two study groups did not differ significantly (*p*’s > 0.05) (Table 1).

Across the entire sample, exactly 50% of the children passed the task. Among children who received standard NICU care following premature delivery, 45% passed the Sally-Anne task. A Chi-square test of independence was performed to examine the relation between study group (FNI versus SC) and performance on the Sally-Anne task. Across the entire sample, the proportion of children who passed the task did not differ between children who received FNI and those who received SC based on an alpha level of 0.05 (χ^2^ (1, *N* = 64) = 0.57, *p* = 0.45) (Table 2).

To determine whether the child’s GA at birth may impact their performance on the task, we divided the children into two GA groups based on the median GA at birth of the study sample: extremely preterm to very preterm (26^0^–30^6^ weeks GA) and very preterm to moderately preterm (31^0^–34^6^ weeks GA). A Chi-square test of independence revealed that performance on the task did not differ between the two GA groups among children who received SC (χ^2^ (1, *N* = 29) = 1.36, *p* = 0.22) (Table 2). However, children who received FNI were more likely to pass the task if they were born extremely preterm to very preterm compared to those who received FNI and were born very preterm to moderately preterm based on an alpha level of 0.05 (χ^2^ (1, *N* = 35) = 8.24, *p* = 0.01) (Table 2).

Within the extremely preterm to very preterm group, 78% (14 of 18) of the children who received FNI passed the Sally-Anne task, whereas only 30% (3 of 10) of the children who received SC passed the task (χ^2^ (1, *N* = 28) = 6.15, *p* = 0.01, effect size = 1.06) (Table 2). Variables that were associated with either the predictor or outcome variables at a significance level of *p* < 0.1 were included in a multiple linear regression model. Based on this a priori criterion, the model evaluated the effect of study group (FNI versus SC) on Sally-Anne task performance (pass or fail) while including the child’s age at testing as a covariate. This model significantly predicted performance on the Sally-Anne task (*F*(2, N = 25) = 4.70, *p* = 0.02), though the study group was no longer significant at *p* < 0.05 (*t*(2, 25) = 1.95, *p* = 0.06). Importantly, children who received FNI were significantly younger at the time of assessment (60.1 ± 0.6) compared to children who received SC (62.5 ± 0.9) (*p* = 0.03). Therefore, the difference between the groups with regard to Sally-Anne task performance is not due the FNI children being older than the SC children at the time of assessment.

Within the very preterm to moderately preterm group, 29% of the children who received SC passed the Sally-Anne task and 53% of the children who received FNI passed the task, though the group difference was not significant (χ^2^ (1, *N* = 36) = 1.99, *p* = 0.16) (Table 2).

As expected, infants born between 26^0^ and 30^6^ weeks GA experienced longer hospital stays in the NICU compared to those born between 31^0^ and 34^6^ weeks GA regardless of study group assignment (*t*(61) = 6.03, *p* < 0.01). The length of stay in the NICU was nearly 40% longer for infants born extremely preterm to very preterm (65 days on average) compared to those born very preterm to moderately preterm (28 days on average). Performance on the Sally-Anne task was not impacted by length of stay among children who received SC. In contrast, the length of stay in the NICU was significantly longer for children who received FNI and who passed the Sally-Anne task (*t*(32) = 11.57, *p* <0.01).

## 4. Discussion

A primary aim of this study was to evaluate the development of ToM in a sample of children who were born prematurely and to determine whether a parental intervention in the NICU could mitigate the previously reported deficits in ToM. We found that 45% of the children who were born prematurely and who received standard NICU care passed the Sally-Anne task, a false belief task designed to assess a child’s ToM abilities. This low pass rate is consistent with a previous study of preterm infants [25] and is lower than the expected pass rate of 55%–80% in full-term infants assessed between 4 and 6 years of age [38,39]. Several studies have evaluated whether the developmental trajectory of social-cognitive skills differs between children born prematurely and those born full-term. Together with our findings, these studies support the notion that children who are born prematurely experience lower social competence throughout childhood and into adolescence [21,22,40,41,42]. Within the first 12 months, infants born prematurely already exhibit deficits in basic social-cognitive skills that are critical for the development of more advanced social-emotional skills including ToM. For example, preterm infants are less likely to initiate joint attention with others, and they also respond to the initiation by others less often than full-term infants [43,44]. Since joint attention contributes to the quality of social interactions between the infant and others, deficits in this basic skill may shape early dyadic interactions and later development of complex social-cognitive abilities such as ToM, which are important for establishing meaningful interpersonal relationships. Therefore, studying early emerging indicators of altered social-cognitive development among children born preterm may guide our understanding of the long-term disruptions in peer relationships that have been observed in these individuals.

Across the total sample included in this analysis, we did not find a significant difference in ToM performance between infants who received FNI and those who received SC during NICU hospitalization. This contrasted with our hypothesis that infants who received FNI in the NICU would perform better on the ToM task. As a secondary analysis, we sought to determine whether GA at birth was associated with performance on the ToM task by comparing performance of infants born extremely preterm to very preterm (26^0^–30^6^ weeks GA) to those born very preterm to moderately preterm (31^0^–34^6^ weeks GA). We expected that ToM deficits would be most evident among infants born at very young GAs. However, among infants who received SC, we did not find an effect of GA at birth on ToM. In contrast, infants who received FNI and who were born extremely preterm to very preterm performed significantly better on the ToM task compared to those born very preterm to moderately preterm. The findings for both SC and FNI infants were contrary to our hypothesis that infants born at a younger GA would experience greater ToM impairments. Further, we only observed group difference (FNI versus SC) in performance when comparing infants who were born extremely preterm to very preterm. One explanation for this unexpected result is that GA at birth is tightly associated with length of stay in the NICU, which in turn is associated with the duration of exposure to FNI. On the other hand, infants born extremely preterm to very preterm who received SC were, on average, exposed to the adversities of the NICU environment for a longer duration than those born very preterm to moderately preterm. Therefore, the FNI infants in this gestational group may have had the protective benefit of prolonged FNI, whereas SC infants were subjected to extended exposure to the standard NICU environment.

This study has several limitations. First, the attrition at the 60-month assessment time point was substantial (~50%), which raises the possibility that the study sample may not be representative, although the demographic characteristics for these infants were similar to those of the entire enrollment cohort [37]. Secondly, despite power analyses suggesting adequate power (0.77) to detect a significant effect of FNI on ToM abilities among the youngest infants and an effect size of 1.06, the sample size of the sub-analysis is small. Third, it is important to consider that the Sally-Anne task requires certain developmental abilities that may be independent of ToM, such as receptive and expressive language [45]. It has also been demonstrated that modifying the conditions and presentation of the task can improve performance in young children and in children with neurodevelopmental disorders [46,47]. Finally, there is potential for confounding variables that we are unable to address in the present analysis. For example, we have limited information regarding the post-discharge at-home environment as well as objective measures of executive function, which may influence theory of mind abilities at 60 months of age [48].

FNI is based on the theory that infant brain development is tied to autonomic co-conditioning mechanisms [49] and that one’s ability to understand and empathize with what others are thinking and feeling (e.g., ToM) is contingent upon early autonomic socio-emotional learning, which begins between mother and fetus in utero and continues following birth. Studies of autonomic function in infants included in this RCT have shown significant improvement in the FNI group by term age and by 5 years [50,51].

Theoretical assumptions of FNI include the notion that the ability to acquire affective empathy—as distinguished from cognitive empathy—following birth is the result of visceral learning (e.g., functional Pavlovian conditioning), as opposed to cognitive learning (e.g., operant conditioning) [49]. The prevailing theories on how to improve ToM to support the use of various forms of operant conditioning that intervene in conscious cognitive processes [52], though our results suggest that functional Pavlovian conditioning may be a particularly advantageous approach in the context of premature birth. Further, FNI may be particularly beneficial to the youngest and most vulnerable prematurely born infants.

Our findings suggest that children born very prematurely who experience a high-nurture environment during NICU hospitalization through the implementation of FNI demonstrate improved ToM abilities at preschool age. It has been previously reported that FNI significantly reduced risk for ASD at 18 months of age [14]. Robust differences in neonatal EEG measures including absolute power [29], cortical functional connectivity and coherence [53], and integrated information indicators of conscious state [54] between the FNI and SC groups. Future studies will be aimed at elucidating the mechanisms by which FNI affects brain development of preterm infants.

In addition, the Nurture Science Program is currently involved in disseminating FNI as the standard of care in two diverse NICU settings in the United States, one in New Jersey and one in Texas. Our efforts to more widely integrate FNI into standard NICU care involve training of NICU staff in the theory and practice of the intervention and establishing advocates to promote the implementation of FNI.

Given the robust association between ToM and social competence across the lifespan, the effect of FNI on ToM abilities has the potential to attenuate risk for later social impairments associated with premature birth.

## Figures and Tables

**Table 1:** Demographic and clinical characteristics.

	Total (*n* = 64)	SC (*n* = 29)	FNI (*n* = 35)		
	Mean (SD)	Mean (SD)	Mean (SD)	*t*	*p*
Maternal Age at Enrollment	34.7 (6.0)	34.9 (4.8)	34.5 (6.9)	0.29	0.77
Gestational Age at Delivery (weeks)	31.2 (2.1)	31.6 (2.2)	30.6 (2.1)	1.88	0.07
	N (%)	N (%)	N (%)	*χ^2^*	*p*
Maternal Race/Ethnicity					
Black/African American	12 (18.7)	7 (24.1)	5 (14.3)	4.96	0.29
Hispanic	16 (25.0)	8 (27.6)	8 (22.9)		
White	28 (43.8)	12 (41.4)	16 (45.7)		
Not Disclosed	8 (12.5)	2 (6.8)	6 (17.1)		
Maternal Education					
High School	5 (7.8)	2 (6.9)	3 (8.6)	0.48	0.79
Some College	7 (10.9)	4 (13.8)	3 (8.6)		
Graduate School	52 (81.3)	23 (79.3)	29 (82.9)		
Child Sex is Male	29 (45.3)	10 (34.5)	19 (54.3)	2.51	0.11
Cesarean Delivery	45 (70.3)	18 (62.1)	27 (77.1)	1.73	0.19

**Table 2:** Main effects of gestational age at birth and study group assignment on task performance.

	% Passed (*n* Passed/*n* Tested)	% Passed (*n* Passed/*n* Tested)	χ^2^	*p*
**Main Effect of GA at Birth**	**26–30 Weeks**	**31–34 Weeks**		
Combined Study Groups	61 (17/28)	42 (15/36)	2.29	0.10
Standard Care	30 (3/10)	53 (10/19)	1.36	0.22
Family Nurture Intervention	78 (14/18)	29 (5/27)	8.24	0.01
**Main Effect of Study Group**	**Standard** **Care**	**Family Nurture Intervention**		
All Gestational Ages	45 (13/29)	54 (19/35)	0.57	0.45
26–30 Weeks	30 (3/10)	78 (14/18)	6.15	0.01
31–34 Weeks	53 (10/19)	29 (5/27)	1.99	0.16

## Data Availability

The data that support the findings of this study are available on request from the corresponding author. The data are not publicly available due to privacy or ethical restrictions.
